# Aircraft Position Estimation Using Deep Convolutional Neural Networks for Low SNR (Signal-to-Noise Ratio) Values

**DOI:** 10.3390/s25010097

**Published:** 2024-12-27

**Authors:** Przemyslaw Mazurek, Wojciech Chlewicki

**Affiliations:** Department of Signal Processing and Multimedia Engineering, West Pomeranian University of Technology in Szczecin, al. Piastow 17, 70-310 Szczecin, Poland

**Keywords:** machine vision, air surveillance, deep convolutional neural networks, image processing

## Abstract

The safety of the airspace could be improved by the use of visual methods for the detection and tracking of aircraft. However, in the case of the small angular size of airplanes and the high noise level in the image, sufficient use of such methods might be difficult. By using the ConvNN (Convolutional Neural Network), it is possible to obtain a detector that performs the segmentation task for aircraft images that are very small and lost in the background noise. In the learning process, a database of actual aircraft images was used. Using the Monte Carlo method, four types of Max algorithms, i.e., Pixel Value, Min. Pixel Value, and Max. Abs. Pixel Value, were compared with ConvNN’s forward architecture. The obtained results showed superior detection with ConvNN. For example, if the standard deviation equals 0.1, it was twice as large. Deep dream analysis for network layers is presented, which shows a preference for images with horizontal contrast lines. The proposed solution uses the processed image values for the tracking process with the raw data using the Track-Before-Detect method.

## 1. Introduction

Airspace safety is an important aspect of protection systems. These systems employ specialized air traffic control measures that utilize primary and secondary radars, operating effectively regardless of weather conditions. With secondary radars, such as the ADS-B system, it is possible to obtain information about the position of an object in space, its identifier, and additional details [1]. Data from the ADS-B system can be verified using information from the primary radar; however, this verification does not always allow a comprehensive assessment of many important parameters. For example, a different aircraft or UAV (Unmanned Aerial Vehicle) might transmit inaccurate data.

Weather radars have demonstrated their effectiveness in detecting airborne targets. The information gathered from this method could be valuable for sharing with air traffic control systems, as highlighted in [2]. Additionally, recent research has focused on shape modeling, which offers useful functionalities for target detection in air traffic control and defense systems [3]. Analyzing weather change characteristics offers important information for air traffic control systems, and this information can also be utilized for aircraft detection [4].

However, data verification can also be achieved by optical means, using position recognition systems, and by identifying the type of aircraft [5]. Monitoring airspace in the optical range can be challenging due to varying atmospheric conditions [6]. However, these systems can operate nearly continuously in certain regions due to favorable weather. Providing this additional information to airspace security systems is valuable as it helps address various potential threats, including terrestrial attacks and asymmetric aggression from other countries.

There are many other methods for the optical detection of airplanes. Most of these methods focus on detecting airplanes present at airports based on satellite images [7,8]. There are also works related to tracking systems, including vision systems. In certain studies, airplanes are represented as dot images or images made up of only a few pixels, meaning they are very small compared to the overall size of the image. This situation necessitates the development of complex methods, which often involve the use of neural networks at various stages. In some instances, the application of neural networks is combined with a multiscale approach [9,10]. In other cases, advanced edge detection techniques are employed [11]. On the other hand, visual classification was used in other works related to airspace safety. These works concern the detection and identification of UAVs [12,13,14,15] and birds [16]. There are also classification methods based on Jet Engine Modulation (JEM) of the primary radar signal [6,17].

Multimodal data fusion could be utilized in tracking systems, such as ADS-B, as an additional data source or for controlling an ALT-AZ camera head. However, this capability is often disabled or limited, particularly for non-civil or intruder aircraft.

This study aimed to develop and evaluate a new methodology for estimating aircraft positions using deep convolutional neural networks. Specifically, it presented a comparative analysis of four different algorithms for position estimation, focusing on the proposed solution based on a deep neural network with a forward architecture. A Monte Carlo simulation was performed to evaluate the efficacy of the algorithms against a backdrop of real images subjected to synthesized noise. Subsequent sections elaborate on the database utilized and the methodology implemented for the extraction of a training set from this database. Furthermore, a Convolutional Neural Network (ConvNN) integrated with data augmentation techniques was proposed to enhance performance. The ensuing sections present the results of the experiments, accompanied by an in-depth discussion. The final chapter summarizes the conclusions drawn from the study and proposes ways for future research.

The following sections describe the database and the method of obtaining a training set using that database. A ConvNN network with data augmentation was also proposed. In the following sections, the results are presented, and a discussion is held. In the Section 6, the conclusions and the further planned work are presented.

## 2. Data

**Figure 1:**
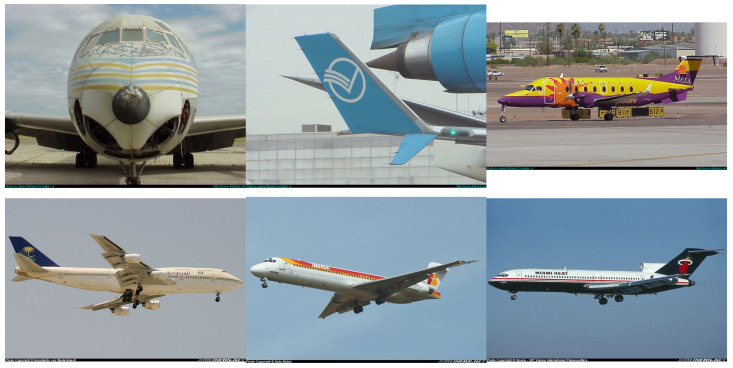
Sample images from the ‘FGVC–Aircraft Benchmark’ database. The images in the upper row represent the rejected images, whereas those in the lower row represent the accepted images in this study.

In this study, the subset of the image database ‘FGVC–Aircraft Benchmark’ [18] that contains various photos of airplanes was used. The pictures of the airplanes were photographed both in flight and at the airport, in different view variants. Atypical and incomplete photos with other background objects, two airplanes, etc., were removed from this database. This was done algorithmically and then with the help of further expert verification. The original database is the database of color images, as shown in (Figure 1). The rejected images are presented in the top row, and the bottom row shows the images accepted for further processing. The issue with the FGVS database lies in the quality of the images. These images do not depict real detection scenarios, where the aircraft is viewed from a considerable distance, leading to a smaller representation in the camera’s frame and a loss of visible details. Factors such as weather conditions and lighting must also be taken into account in these situations, additionally.

This database serves as an appropriate tool for evaluating algorithms in general. However, it is important to note that its applicability to practical scenarios, specifically in the realm of detecting aircraft in flight, is limited. The FGVS database has been used for many years as a benchmark [19], achieving over 96% accuracy using the best solutions. However, this high accuracy is only applicable to very high-resolution, noise-free color images. Therefore, these results do not represent real-world applications related to aircraft detection under actual conditions.

The subset prepared in this way contained 1536 photos (available on github [20]), which were scaled to the resolution of 64×64 so that the airplane had a horizontal or vertical size of 64 pixels. The photos were processed in such a way as to remove clouds from the background. The size of airplanes is between 3 and 16 pixels (length), so they start from the small object class (about 10 pixels occupied) and cover the extended object class (less than 100 pixels). The small object class is very difficult to detect and requires tracking using a video sequence [6]. In the case of the extended object class, detection is possible using a single frame under good visibility conditions because multiple pixels cover information about the object, and there is a possibility to use knowledge about shape and reflected light from airplanes. The camera or object motion blur is less important because a distant object moves with small angular velocity. Atmospheric scintillation is important for distance detection [21]. In the domain of object tracking, the implementation of image distortion models that account for various atmospheric phenomena can be utilized. Although techniques such as active camera cooling can mitigate image sensor noise, it is important to acknowledge that such noise remains an inherent characteristic of image acquisition systems [22].

In the process of augmenting the images, the following operations were performed: real-time data augmentation left–right flip, angle ±5 deg, and scaling the contrast so that the background was 0 and the maximum luminescent value was +1 or −1, respectively; Gaussian noise was added with a zero mean value and variable standard deviation (0–0.1). The noise level value ranged from 0 to such a large value that the airplane was no longer visible to a human. The size of the airplane was changed from 16 pixels (the base image was 64 pixels) to 4 pixels.

This study did not test the ability to classify airplanes, but only the ability to detect airplanes based on a single image frame. Most of the airplanes were viewed from the side or from below. The samples with condensation streaks have been removed from the database. The image of an airplane taken during the day depends on the orientation and position of the airplane in 3D space towards the observer and the sun.

In this investigation, grayscale images were used. The database consisted of images with various lighting configurations. Because the reference point is the background, normalized to the value 0, the darker (usually darkened and dark-painted) values are negative, and the rest are positive. Detection of the airplane in the image was not based on the areas with pixel values above level 0 (bright parts of the airplane) but on both light and dark areas and the spatial relations between them. Many civil aircraft are painted in bright colors such as red. In contrast to them, military airplanes use camouflage in the form of painting in the color usually gray. The database contains a mask specifying the location of the airplane’s pixels and the background, which was used to compare the methods.

## 3. Method

**Figure 2:**
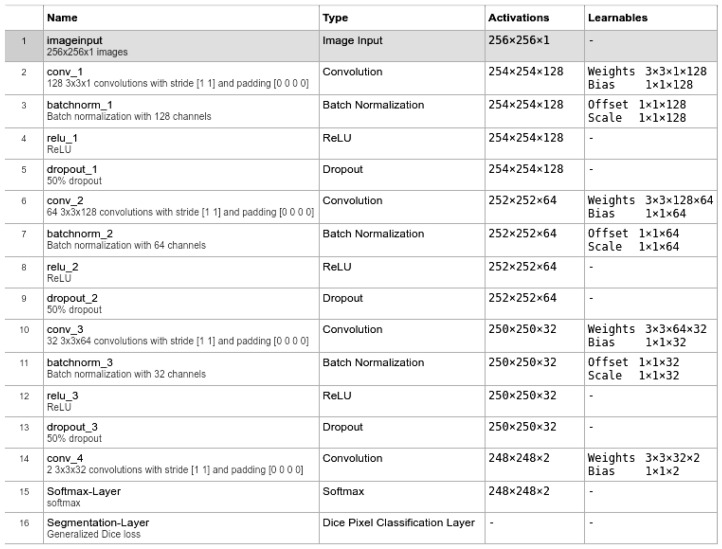
Example configuration of the ConvNN architecture 3×3 128-64-32.

There are many different methods for detecting objects in an image [23,24,25]. In the case of strong noise (small signal-to-noise ratio), the detection of objects is not possible based on a single pixel, and it is necessary to use spatial information. This can be done theoretically with a 2D matched filter bank, but such a bank must be very large due to different shapes, orientations, scales, and lighting conditions. For this reason, the use of neural networks seemed to be more effective due to the much smaller scale of filters and the transition from shallow to deep architecture. In this work, a ConvNN was used, with a typical forward deep architecture. Exemplary configuration of the ConvNN architecture is shown in the Figure 2.

Different configurations were tested, as presented in Table 1. Two sizes of convolutions were considered, i.e., 3×3 and 5×5, for various numbers of neurons, respectively. Four convolutional layers were applied with two main configurations. In the first configuration, successive reduction of the number of neurons in successive layers was assumed. The secondary configuration assumed a fixed number of neurons, and only the last layer used the two neurons.

The neural network underwent training under multiple configurations. The solution of the network learned over 5000 epochs was chosen. The images were grouped 64 in one to take advantage of the features of the convolutional network. The resulting network operated as a semantic network with two output classes (background/sky and airplane). The training process was facilitated using an NVIDIA GeForce GTX Titan X (Santa Clara, CA, USA) and NVIDIA Quadro RTX 8000 graphics processing units in conjunction with Matlab R2024b, specifically using the Deep Learning Toolbox [26]. The real-time augmentation process described in Section 2 was used.

As shown in Table 2, the learning algorithms used were: Stochastic Gradient Descent with Momentum (SGDM) [27], RMSpror, and ADAM [28].

## 4. Results

**Figure 3:**
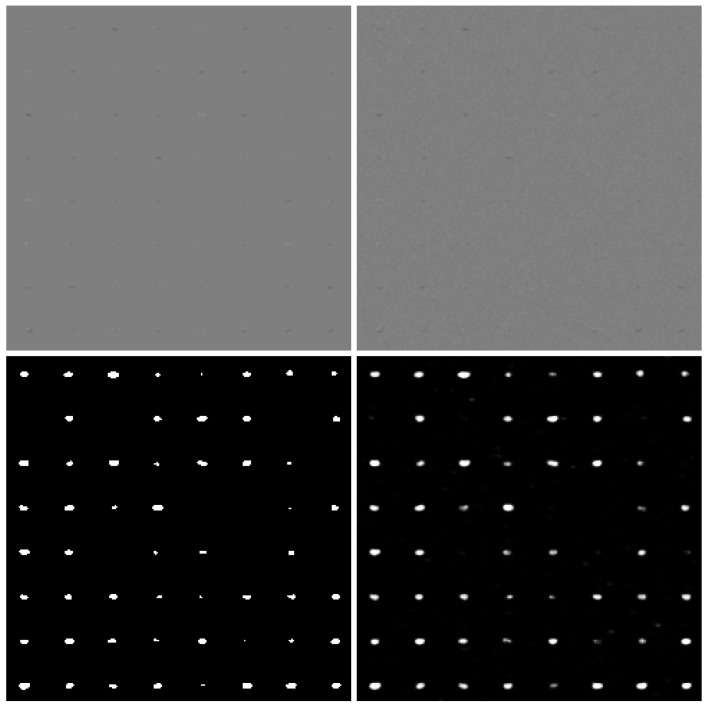
Exemplary results for small airplanes. Noiseless (**left–top**) reference, noised image (std.dev. = 0.02) (**right–top**), binary output decision (**left–bottom**), and detection values (**right–bottom**).

**Figure 4:**
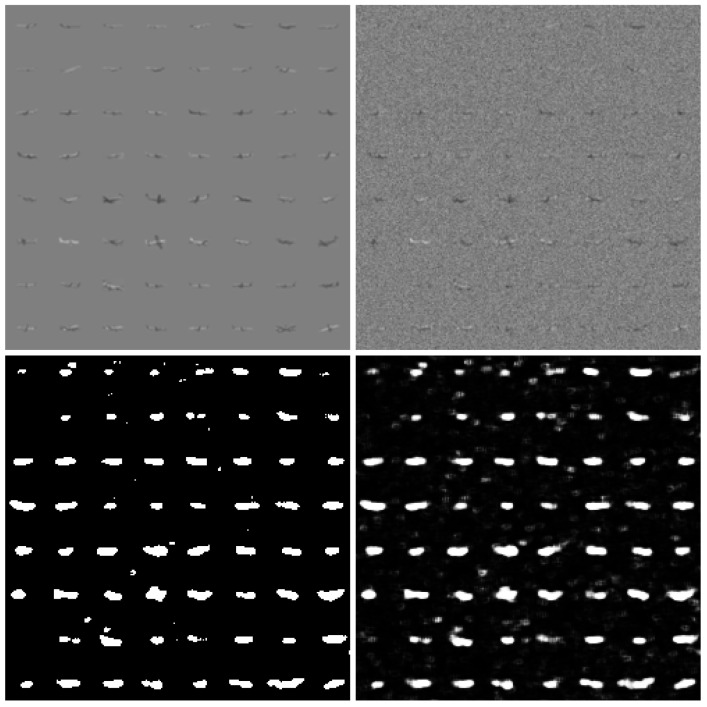
Exemplary results for large airplanes. Noiseless (**left–top**) reference, noised image (std.dev. = 0.10) (**right–top**), binary output decision (**left–bottom**), and detection values (**right–bottom**).

In testing, image blocks with a size of 32×32 pixels and a corresponding mask for the airplane’s pixels were used. The resistance test of detection algorithms for the Monte Carlo method was utilized in this process. Gaussian noise was used, with the standard deviation ranging from 0 to 0.5 with a step of 0.01. A total of 153,600 of aircraft images were used for each noise level. Sample ConvNN image responses are shown in Figure 3 and Figure 4.

**Figure 5:**
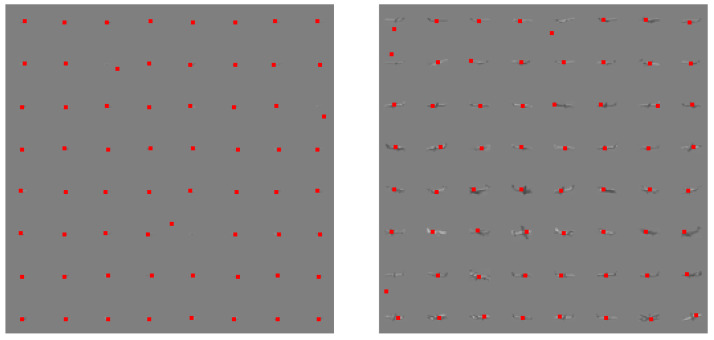
Exemplary reference images and detections (red markers) based on the maximal value response of ConvNN.

The basic criteria are the following measures: Max. Pixel Value, Min. Pixel Value, and Max. Abs. Pixel Value for pixel values related to the airplane area (mask) relative to the background. Since the airplane can have brighter and darker pixels compared to the background, it can be detected in three ways by a local algorithm (moving window) using one of these criteria. The maximum absolute value search criterion is more universal than the first two. Exemplary reference images and detections based on the maximal value response of ConvNN are presented in Figure 5.

**Figure 6:**
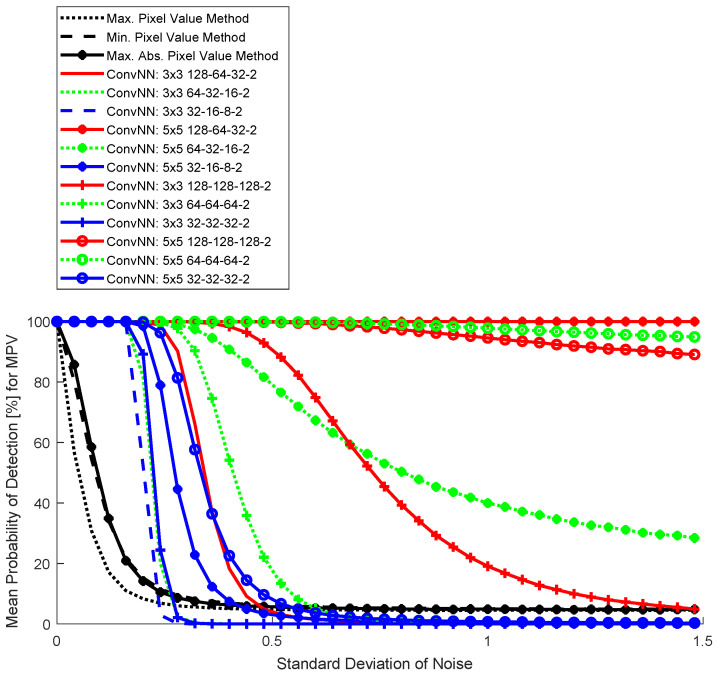
Monte Carlo results for four detection algorithms: trivial (Max, Min, and Max Abs) and convolutional neural networks (SGDM).

**Figure 7:**
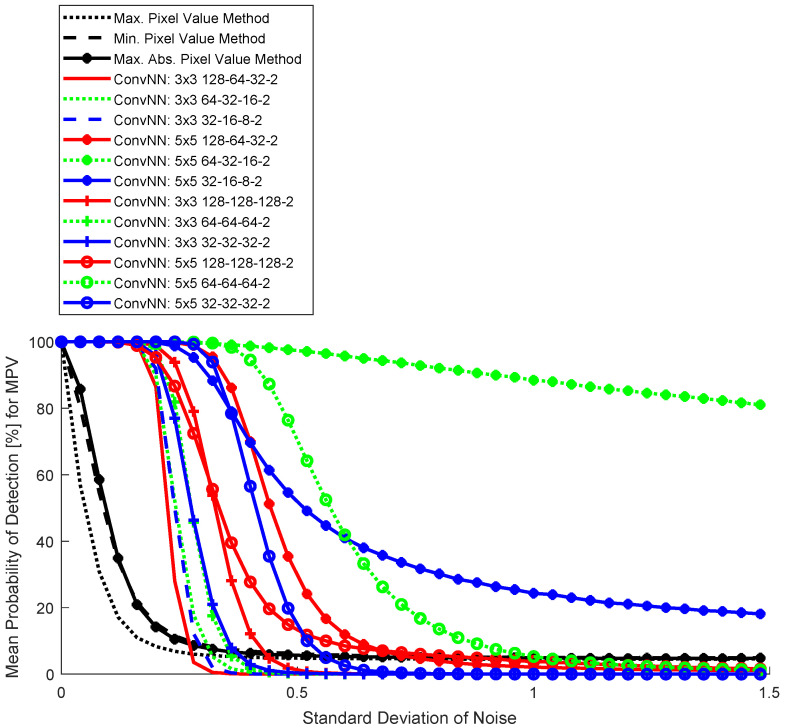
Monte Carlo results for four detection algorithms: trivial (Max, Min and Max Abs) and convolutional neural networks (RMSprop).

**Figure 8:**
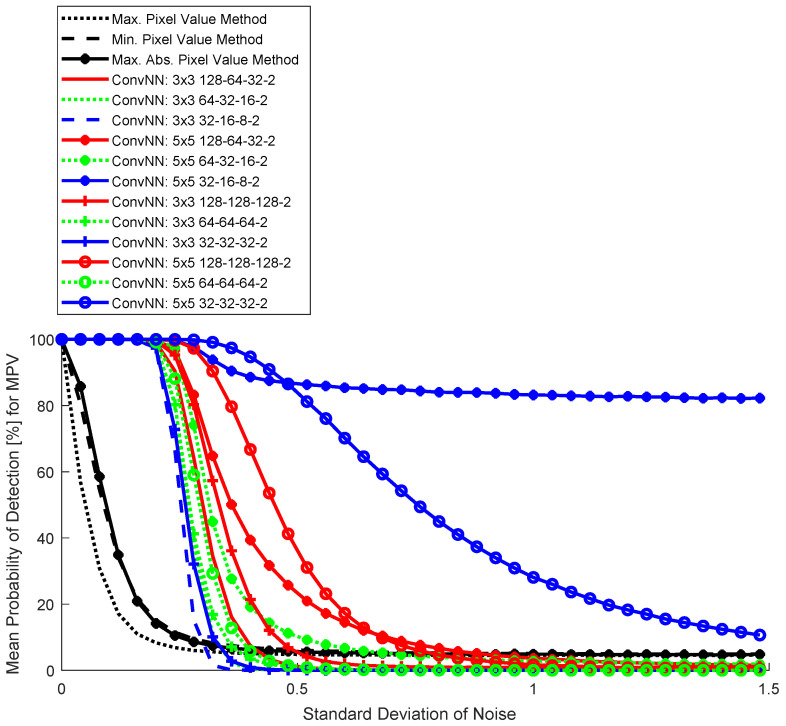
Monte Carlo results for four detection algorithms: trivial (Max, Min, and Max Abs) and convolutional neural networks (ADAM).

The results for the three basic detection methods and ConvNNs are shown in dependence on the level of Gaussian noise in Figure 6 (SGDM training algorithm), Figure 7 (RMSprop training algorithm), and Figure 8 (ADAM training algorithm). For simplicity of visualization, the results are noise-changed in the steps of 0.03. The results for basic detection algorithms using pixel values are shown in black, and red for ConvNNs where the first convolutional layer contains 128 neurons, green 64 neurons, and blue 32 neurons. All ConvNNs achieved after 5000 steps mini-batch accuracy >97%.

**Figure 9:**
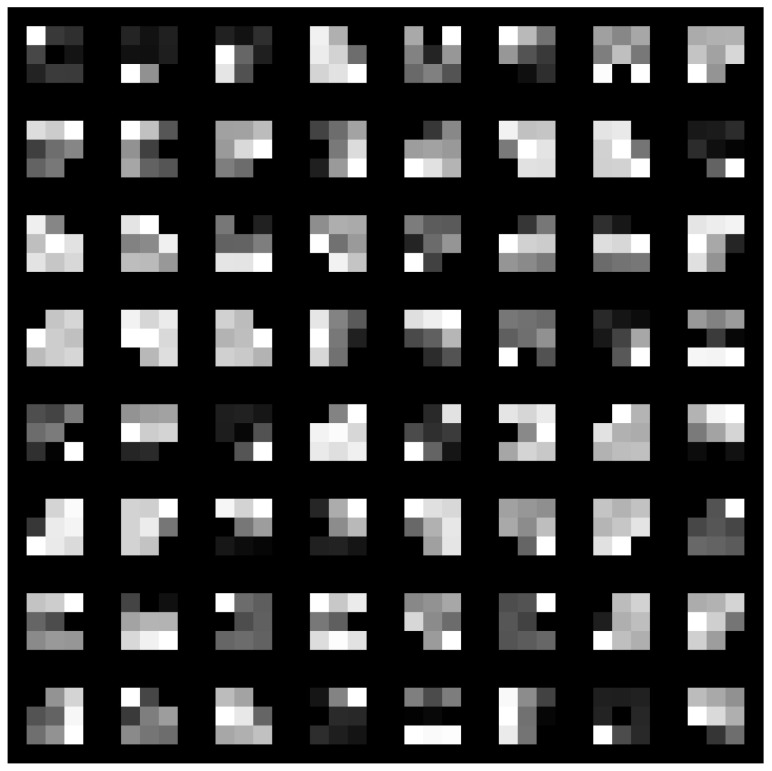
Image of the weight mask associated with the first convolutional layer of the neural network specified in row 2 (3×3 64-32-16) of Table 1.

**Figure 10:**
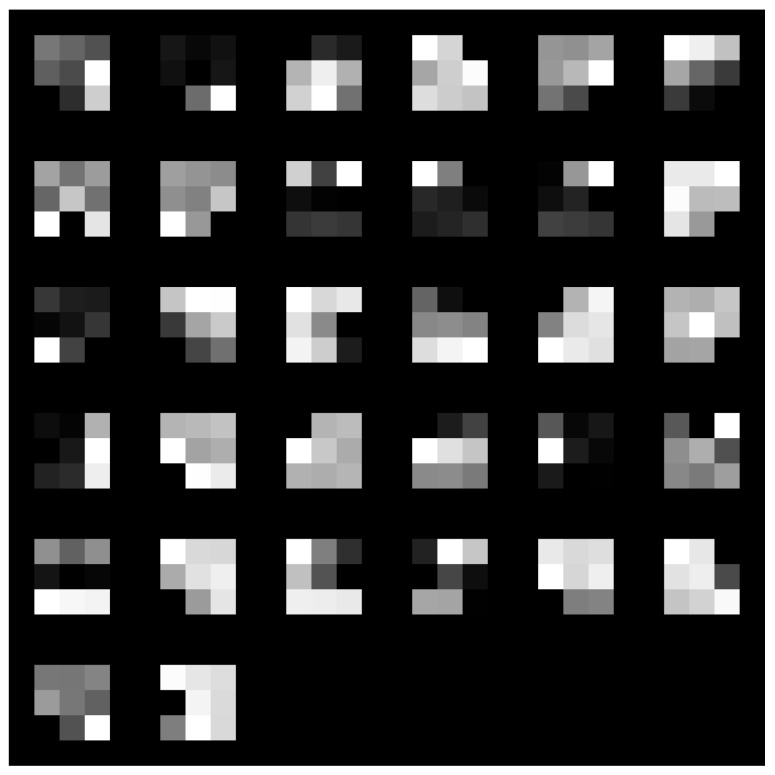
Image of the weight mask associated with the first convolutional layer of the neural network specified in row 3 (3×3 32-16-8) of Table 1.

**Figure 11:**
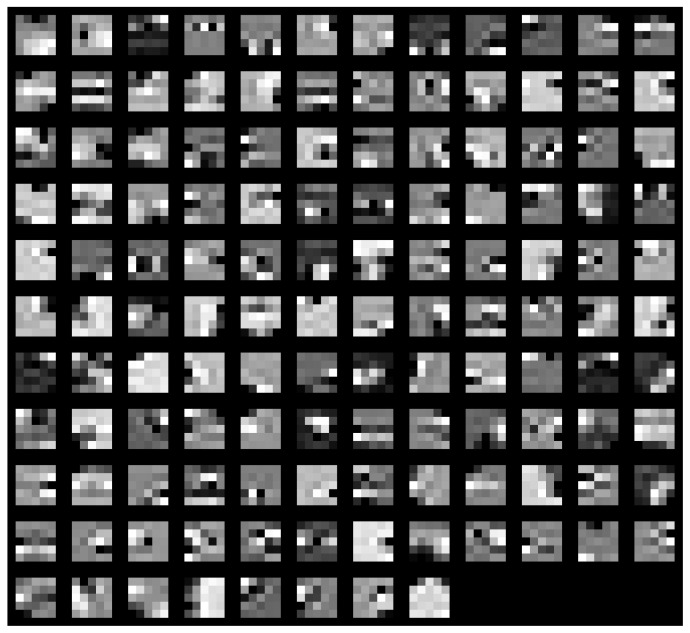
Image of the weight mask associated with the first convolutional layer of the neural network specified in row 4 (5×5 126-64-32) of Table 1.

**Figure 12:**
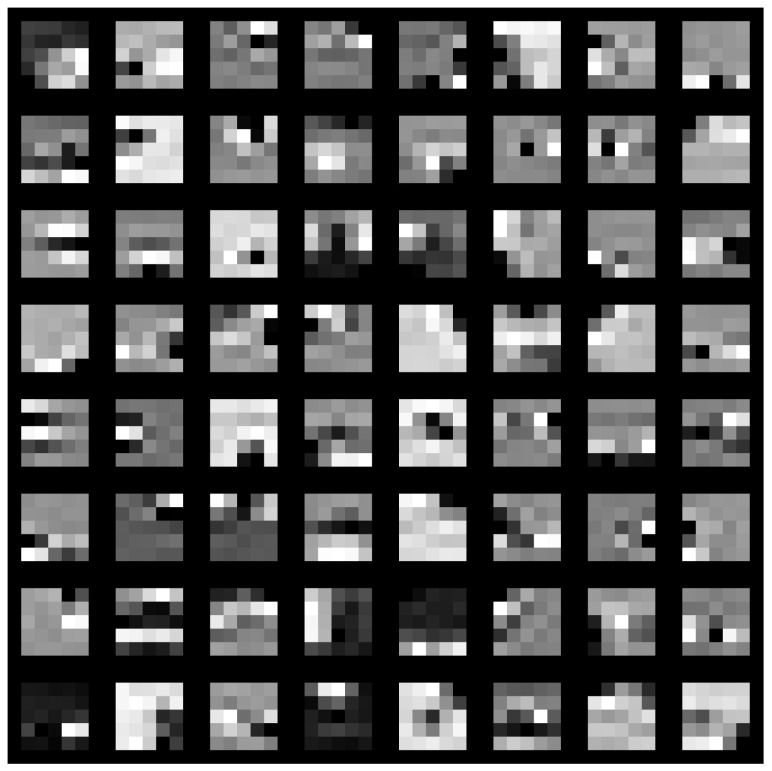
Image of the weight mask associated with the first convolutional layer of the neural network specified in row 5 (5×5 64-32-16) of Table 1.

**Figure 13:**
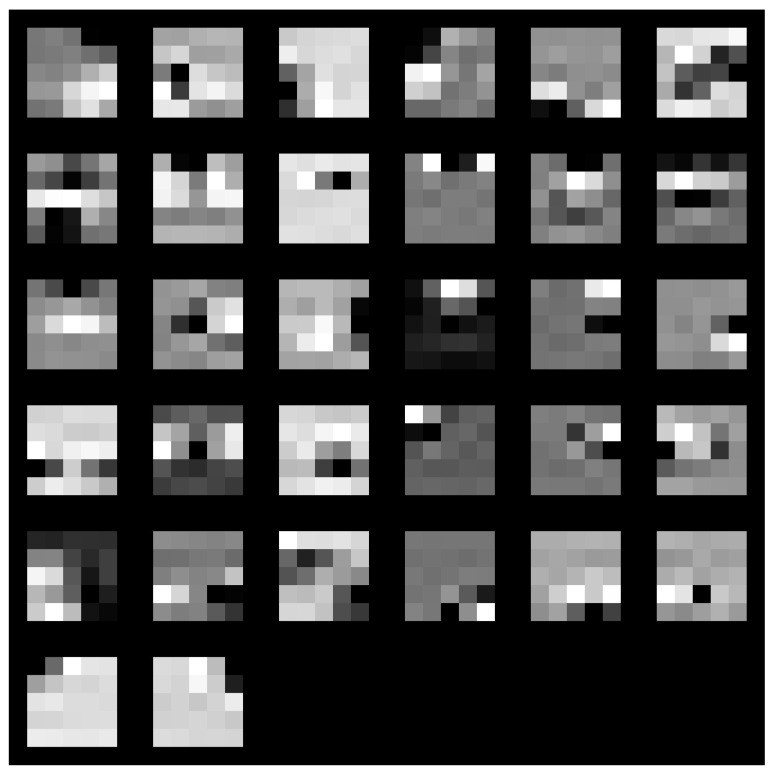
Image of the weight mask associated with the first convolutional layer of the neural network specified in row 6 (5×5 32-16-8) of Table 1.

Two distinct visualization methods can be employed for individual cases to ascertain the learning patterns of a neural network. The first approach involves visualizing the weights originating from the first layer of the neural network [29]. It is commonly anticipated that these weights function as shape detectors (see Figure 9, Figure 10, Figure 11, Figure 12 and Figure 13), as mentioned by the existing literature [30,31,32]. The second approach involves a technique that enables the visualization of individual neuron activations within the network, commonly called deep dream, as referenced in [33,34].

Using a detector in the form of a ConvNN maximizes the value for the airplane (they are larger than the background) because the expected output in the learning process is the mask. This allows the estimation of a very approximate silhouette region of the aircraft. Using the maximum value search algorithm is more efficient because it is not related to one pixel, but is the result of larger pixel group relationships. The semantic network returns two numerical results for each pixel, corresponding to the degree of belonging to each class. A value comparison was used to obtain a detection with binary output.

The network training time is shown in Table 3 for the NVIDIA Quadro RTX 8000 graphics processing unit.

## 5. Discussion

The Monte Carlo method, using real images and controlled disturbances, allowed for the estimation of the algorithm’s potential. The advantage of an approach with a detector that is learned by various luminance configurations is independence from sudden changes in lighting. For example, the use of the sliding window correlation method for successive frames is very difficult in such cases.

Location detection with the proposed ConvNN is very effective (especially for Figure 6). In the absence of noise (which is very difficult to obtain, in fact, due to the noise associated with the acquisition process or atmospheric fluctuations), a trivial algorithm could be used that, based on the estimated background, detracts the signal from the camera. Detection of very small airplanes, even with the size of one pixel, is then possible. This is not possible in a realistic case with image noise, and a machine learning approach is required.

In the 32×32 blocks, the largest pixel value was related to the airplane area (Figure 5); therefore, the curve for ConvNN differs significantly from other simple algorithms (Figure 6, Figure 7 and Figure 8). This does not mean, however, that the high values are not in the background area, but the local value for the airplane is dominant. It is not appropriate to use detection by comparing both responses from a semantic network (which is binary). This leads to a large number of false detections in the background area, therefore this type of analysis was abandoned. This problem is well known in the literature related to tracking objects with a signal similar to background noise [35].

The correct approach is based on the rejection of the binary response from the aircraft detector in favor of tracking the signal (Figure 5) for the given trajectories. This method is known as TBD (Track–Before–Detect) [36,37,38,39,40] and requires very high computing power because all possible trajectories have to be tracked on the measurement sequence (in this case, video sequence) [36,41,42,43,44]. This tracking increases the SNR (Signal–to–Noise Ratio), thanks to which effective detection is possible only after that.

The best result was achieved by a network with a 5×5 convolution window and an architecture with a successively decreasing number of neurons in successive hidden layers (128-64-32). In comparison, changing the convolution window to 3×3 resulted in a much worse result. Increasing the number of neurons in subsequent hidden layers (128-128-128) gave a worse result, which can be interpreted as too weak convergence, despite the local accuracy value being >97%. This shows that analyzing this type of network more thoroughly under strong noise conditions during training is necessary. The advantage of a network with a larger convolution window is that the tracked objects are larger than individual pixels and can be easily detected by 2D filtering.

**Figure 14:**
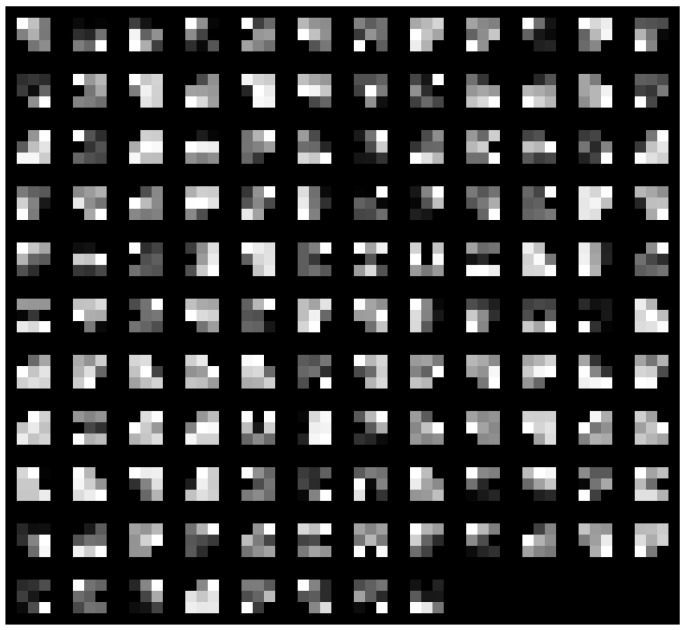
Image of the weight mask associated with the first convolutional layer of the neural network specified in row 1 (3×3 128-64-32) of Table 1.

Based on the analysis of the figures (see Figure 9, Figure 10, Figure 11, Figure 12, Figure 13 and Figure 14), it can be concluded that all examined networks tend to detect fine details within the images. A notable characteristic shared among these networks is the identification of both bright and dark individual pixels. This phenomenon can be attributed to the fact that such pixel values enhance the likelihood of accurately detecting airplanes against a predominantly gray background. Furthermore, another salient feature observed is the detection of horizontal lines and edges. This can be explained by the composition of the dataset, which contains a significant number of aircraft displayed in horizontal or nearly horizontal orientations. This is typical for observing aircraft from a long distance. However, when an aircraft flies close to the camera, different angles can be captured. From a security perspective, detecting aircraft from greater distances is more important, as it allows for shorter reaction times when an aircraft appears, which is crucial for both civil and military applications.

Networks that have a larger number of neurons in the first layer offer a greater variety of feature detectors for images. Likewise, a network that uses a larger mask size (5×5) emphasizes edge features more effectively than one that uses a smaller mask size (3×3).

Larger masks enable the detection of larger features within an image, aligning with the principle of matched filters used for feature detection [45]. Conversely, when a smaller mask is used, the detection of larger features will be deferred to subsequent layers. Using very large masks can result in two scenarios: either mask reduction, where some weights are zeroed out, limiting the effective size of the mask, or the storage of the entire image of the object, such as an airplane. In the latter scenario, if the first layer contains a very large number of neurons, the network may function as a bank of matched filters that potentially perform interpolations. This is disadvantageous if the learning process—specifically the dataset and its augmentation—is not sufficiently diverse. Very large masks with a large number of neurons can result in slower network learning and increased processing costs, especially for real-time applications, additionally.

From a military standpoint, the detection of features associated with camouflage seems to be interesting. Implementing a strategy to minimize contrast in aircraft paint is advisable, with the optimal goal being to achieve a brightness that closely aligns with the surrounding environment. Nevertheless, it is essential to acknowledge that varying lighting conditions can produce shadows, which may lead to the emergence of darker areas that can be effectively detected by neural networks.

**Figure 15:**
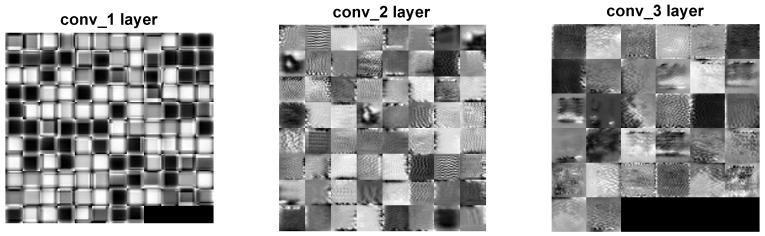
Image of the deep dream feature for convolutional layers of the neural network specified in row 4 (5×5 126-64-32) of Table 1.

**Figure 16:**
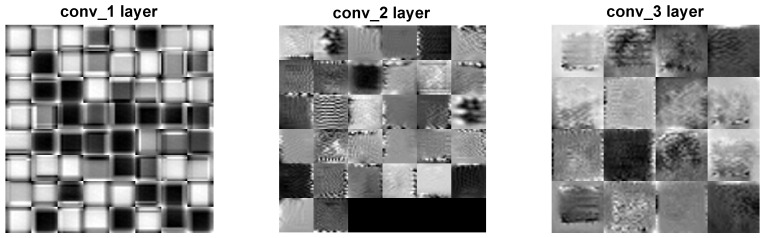
Image of the deep dream feature for convolutional layers of the neural network specified in row 5 (5×5 64-32-16) of Table 1.

**Figure 17:**
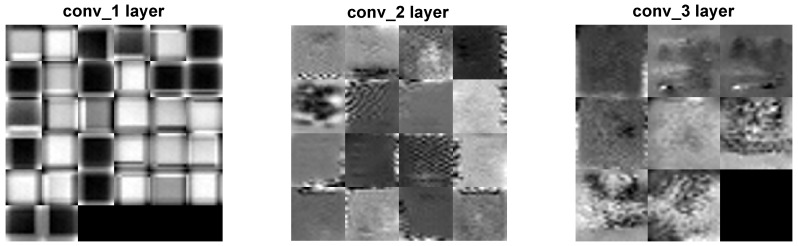
Image of the deep dream feature for convolutional layers of the neural network specified in row 6 (5×5 32-16-8) of Table 1.

**Figure 18:**
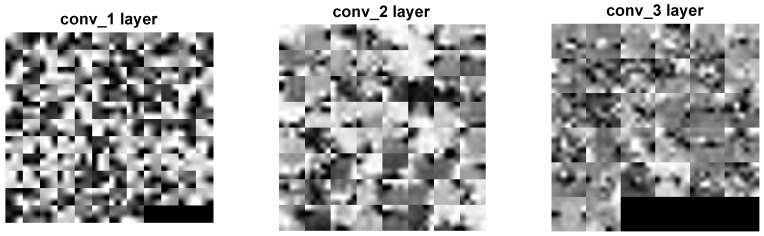
Image of the deep dream feature for convolutional layers of the neural network specified in row 1 (3×3 128-64-32)of Table 1.

**Figure 19:**
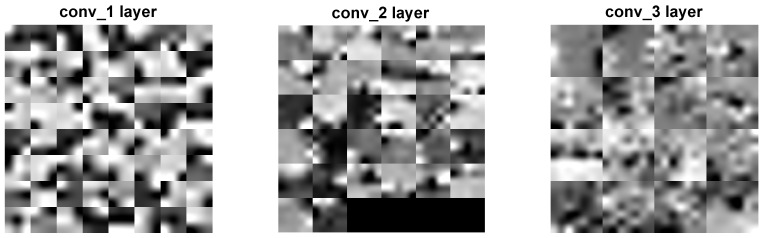
Image of the deep dream feature for convolutional layers of the neural network specified in row 2 (3×3 64-32-16) of Table 1.

**Figure 20:**
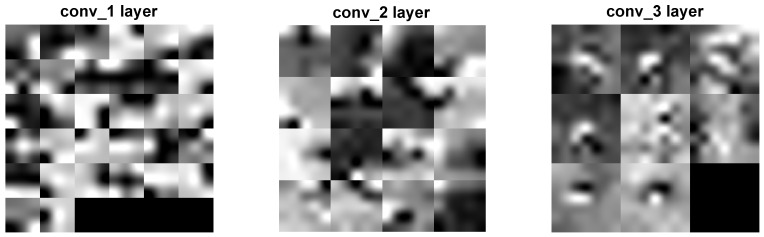
Image of the deep dream feature for convolutional layers of the neural network specified in row 3 (3×3 32-16-8) of Table 1.

The deep dream study [33,34] demonstrates what input images can cause significant activation of neurons. However, this method is not straightforward, and only one sample image is presented. Layer $conv_{1}$ represents the output of the first convolutional mask. In contrast, for the network shown in Figure 15, Figure 16 and Figure 17, the images exhibit lower contrast and feature horizontal lines. In Figure 18, Figure 19 and Figure 20, the shape detectors appear at the very beginning. Regarding the analysis of the second layer (again referring to Figure 15, Figure 16 and Figure 17), the strong activations correspond to raster-like images that also include line rasters. This means that the high-frequency components have a greater share in detection over the entire image area. It includes identifying edges that are either brighter or darker than the background, as well as edges that contain a mix of both bright and dark lines or pixels concerning the background. These characteristics are also observed in the $conv_{3}$ layer of these networks. Other networks exhibit similar features in their $conv_{3}$ layers, although they are not as prominently displayed.

In summary, deep dream analysis offers valuable insights into how networks operate and how they are trained. This suggests the possibility of designing filters intentionally rather than creating detectors solely through training the network. Such an approach could lead to the development of more effective solutions that account for the specificities of signal processing.

This analysis offers critical information regarding both optimal and suboptimal camouflage techniques. In the context of military applications, it further elucidates strategies for flight maneuvers designed to minimize optical detection within the visible spectrum, taking into consideration variables such as solar orientation and cloud cover.

**Figure 21:**
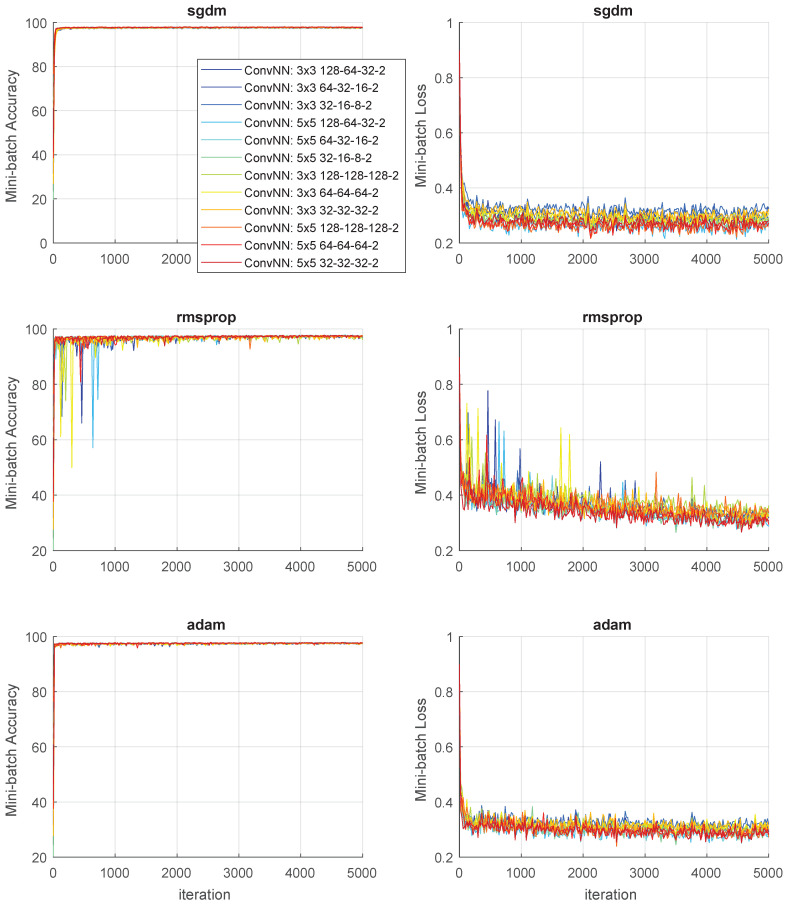
Convergence of algorithms for different ConvNN configurations.

Figure 21 illustrates the convergence process for training data across three different algorithms and various network configurations. The graph demonstrates that the accuracy ratio is quickly achieved within 100 iterations, indicating the strong performance of the deep network for object detection when using the SGDM and ADAM algorithms. However, in the case of the RMSprop algorithm, the accuracy values for subsequent batches fluctuate significantly, necessitating a much larger number of iterations (over 4000) to stabilize the accuracy. Additionally, the mini-batch loss graphs highlight the impact of the chosen convolution mask size. Specifically, smaller masks (size 3×3) correspond to higher error values, while larger masks (size 5×5) and more complex networks yield lower error values.

The training time values presented in Table 3 are relatively short due to the use of images with a larger number of planes for training. This approach also promotes better data balance. As a result, the computational capabilities of the convolutional network on the GPU are utilized more effectively during the training process. This indicates that the proposed network architectures are well-suited for larger systems.

## 6. Conclusions and Further Work

In this work, the detection based on ConvNN was presented with promising results. The advantage of this approach is its scalability for vision systems due to the simple structure using a weave. It can also be used in cameras that use intelligent image processing.

Further work will focus on improving the quality of the estimation of location in cloudy conditions—changing backgrounds and supplementing with tracking algorithms to further improve the quality of position estimation. The use of tracking algorithms [6] might have a positive impact on the improvement of the entire process, especially at high noise levels. Particularly, when the signal could be lost in the background noise and it is not possible to detect it based on one observation, but it is possible based on a series (in this case, video sequences).

Another problem, and at the same time a method of position estimation, is the use of information about condensation trails. Depending on the weather conditions, they are visible or not and could be a very good indicator of the presence of an aircraft also from a very long distance.

## Figures and Tables

**Table 1:** Configurations of tested ConvNN.

No.	Convolution	Conv_1	Conv_2	Conv_3	Conv_4
		[No. Neurons]	[No. Neurons]	[No. Neurons]	[No. Neurons]
1	3×3	128	64	32	2
2	3×3	64	32	16	2
3	3×3	32	16	8	2
4	5×5	128	64	32	2
5	5×5	64	32	16	2
6	5×5	32	16	8	2
7	3×3	128	128	128	2
8	3×3	64	64	64	2
9	3×3	32	32	32	2
10	5×5	128	128	128	2
11	5×5	64	64	64	2
12	5×5	32	32	32	2

**Table 2:** Configurations of tested ConvNN.

Algorithm	SGDM	RMSprop	ADAM
Initial Learn Rate	0.1	0.1	0.1
Regularization	$L_{2}$	$L_{2}$	$L_{2}$
Momentum	0.9	-	-
Learn Rate Drop Factor	0.98	0.98	0.98
Learn Rate Drop Period	50	50	50
Max. Epochs	5000	5000	5000
Mini-Batch Size	150	150	150
Learn Rate Schedule	piecewise	piecewise	piecewise
Shuffle	every-epoch	every-epoch	every-epoch
Gradient Threshold Method	$L_{2}$ norm	$L_{2}$ norm	$L_{2}$ norm
Gradient Threshold	0.05	0.05	0.05

**Table 3:** Learning time for various configurations of the evaluated methods.

No.	ConvNN Configuration	SGDM	RMSprop	ADAM
1	3×3 128-64-32	02:24:45	02:24:40	02:23:57
2	3×3 64-32-16	02:38:54	02:00:59	02:00:59
3	3×3 32-16-8	02:29:15	01:50:33	01:50:31
4	5×5 128-64-32	03:17:58	02:35:43	02:35:08
5	5×5 64-32-16	02:46:17	02:08:37	02:07:32
6	5×5 32-16-8	02:33:34	01:53:19	01:52:45
7	3×3 128-128-128	04:57:54	05:07:36	05:06:19
8	3×3 64-64-64	03:01:41	02:16:43	02:16:20
9	3×3 32-32-32	02:36:08	01:56:17	01:55:04
10	5×5 128-128-128	05:13:55	06:07:06	06:06:12
11	5×5 64-64-64	03:05:48	02:28:50	02:27:06
12	5×5 32-32-32	02:41:30	02:01:17	02:01:01

## Data Availability

Data are available from the authors.

## Abbreviations

The following abbreviations are used in this manuscript:

ADAM	ADAptive Moment Estimation
ALT-AZ	Altitude-Azimuth
ConvNN	Convolutional Neural Network
UAV	Unmanned Aerial Vehicle
JEM	Jet Engine Modulation
SGDM	Stochastic Gradient Descent with Momentum
TBD	Track Before Detect
SNR	Signal To Noise Ratio
